# Dissemination and participation in early warnings and disaster risk reduction in South Africa

**DOI:** 10.4102/jamba.v16i1.1566

**Published:** 2024-01-31

**Authors:** Collins Muhame, Alice Ncube, Yonas T. Bahta

**Affiliations:** 1 Disaster Management Training and Education Centre for Africa (DiMTEC), Faculty of Natural and Agricultural Sciences, University of the Free State, Bloemfontein, South Africa; 2 Department of Agricultural Economics, Faculty of Natural and Agricultural Sciences, University of the Free State, Bloemfontein, South Africa

**Keywords:** metropolitan, non-metropolitan cities, ward committee, disaster preparedness, sustainable human settlement, urban resilience, informal settlement

## Abstract

Governments cannot effectively manage and handle disasters, particularly at the local community level, without actively engaging vulnerable people. The key to achieving sustainability in disaster recovery is community participation and information dissemination. The informal settlements’ lack of access to information and public engagement hampered their ability to recovery, thus prompting this study. Therefore, many cities and intervention partnerships faced information and participation gaps in disaster risk reduction (DRR). The study’s rationale was to determine the participation and communication of Khayalitjha household heads, regarding DRR information dissemination for sustainable human settlement, using a cross-sectional household survey of 295 household heads from Khayalitjha in situ informal settlement in the Free State provinces of South Africa. The security of dwelling unit tenure concept was an indirect indicator used to measure social resilience. The key findings revealed that community volunteers, ward committee members and most of the respondents, were responsible for initiating the DRR and disaster preparedness planning process. This indicated that local government needs to strengthen the human resource capacity building for DRR management information dissemination at a local level. The church, school, WhatsApp, Facebook, Twitter and Instagram were the preferred modes of communication for early warnings of disaster information.

**Contribution:**

Despite advocating for a multidisciplinary stakeholder approach, urban DRR studies tend to ignore communities in high disaster-risk areas. Employing social resilience, it aims to extend the DRR information dissemination strategy to in situ informal settlements beyond the communication and public participation advocacy strategies of local municipal urban cities.

## Introduction

Early warning is defined as:

[*T*]he provision of timely and effective information, through identified institutions, that allows hazard to be used for communicating it to the public, to prepare them for the hazard’s arrival, and to take actions to reduce hazard risk, while the Red Cross and the Red Crescent Societies define an early warning system as the set of capacities needed to generate and disseminate timely and meaningful warning information to enable individuals, communities, and organizations threatened by a hazard to the community. (IFRC, 2012, p. 9)

Communication of uncertainty towards science advice, forecasting, and uncertain communication model, has presented a challenging environment for planning and decision-making among stakeholders, emergency officials, and the greater public (Doyle et al. 2019; Pineda 2015).

A common challenge among urban informal settlements is finding ways to conduct anticipatory disaster risk reduction (DRR) rather than resorting to purely reactionary humanitarian support deployed DRR, including early warning systems (EWS). The EWS for climate-related hazards depends on climate services and the ability to forecast and detect potential hazards. The EWS can assist people and institutions in improving ex ante decision-making and implementing predetermined intervention actions to protect people from disaster impacts (Peters et al. 2022).

Although climate-change adaptation is considered a global phenomenon of natural hazards, the response to climate-induced risks is predominantly at the local level. This renders the local government relevant to mainstreaming DRR into sustainable human settlement. Local governments should focus on implementing policies and strategies that lessen the impacts of climate-induced risks on individuals and communities (Williams et al. 2018). The city officials are expected to have urban resilience plans in place and uncoordinated management styles must give way to integrated partnership efforts that can address morally unacceptable community challenges. Professional expertise should be less centralised and evenly distributed, and the issue of risk reduction can be solved through effective communication. Thus, emphasising knowledge dissemination and sharing responsibilities in multidisciplinary networks, including the latest social communication platforms. This can be achieved by localising DRR activities and linking them with sustainable human settlement and informal settlement upgrading programmes (UN-Habitat (a) 2017) in the communication channels.

Various urban and regional policies were approved post-1994 (Izume et al. 2019). These were policies such as the Reconstruction and Development-Programme (RDP), Integrated-Urban-Development Framework (IUDF), Breaking-New-Ground (BNG) programme, and the New Urban Agenda (NUA). Marais and Visser (eds. 2008) explained that at the centre of these policies, restructuring historic spatial patterns of South Africa was the main objective, while densification and integration of the city transport network characterised the urban policy approaches. However, the BNG policy that was implemented in 2004 did not achieve the intended outcome of addressing housing backlogs (Izume et al. 2019). As a result, in situ informal settlements of the Free State Provinces of South Africa face both existing and future stresses and risks because of climate change. This then calls for building urban resilience within sustainable human settlement and in situ informal settlement upgrading.

A significant challenge for DRR intervention measures is a lack of a thorough understanding of the urban in situ informal settlement context of DRR. This is both in terms of the urban societal structures and urban profiles of the communities (IFRC 2017). The eradication of informal settlements and the delivery of RDP houses would not address the informal settlement backlogs alone. This is because they also produce more negative unintended consequences, such as relocations of communities and a loss of people’s livelihoods (RSA HDA (b) 2014). The one significant contributing factor to the slow pace of housing delivery is insufficient budget and partly the escalating construction costs (Mukorombi 2014).

The Sendai Framework for Disaster Risk Reduction (SFDRR) is also silent on building capacities of local, regional governments, and integrating DRR within local regulatory bi-laws, legal policy frameworks, and land-use management legislations. Regarding low-income earners residing within urban cities’ informal settlements and the contribution of civil societies towards building urban resilience, the SFDRR also exhibits some weakness in essential measures for mainstreaming DRR effectively within informal settlement upgrading (IIED 2019). The DRR and management framework proposed for urban cities must contribute accurately and globally aligned indicators to best support local and national government’s efforts towards building urban resilience for in situ informal settlement upgrading.

Most disasters that vulnerable communities at informal settlements face are recurrent, small-scale disasters that neither trigger media or government attention nor attract external disaster relief aid support (USAID 2019). This is further highlighted in the National Treasury Cities Support Program report, stating that there are serious gaps in the coverage and quality of spatial lower geographic level of cities’ socio-economic data in South Africa. The current gap of missing data on local and regional cities became a handicap for government policymakers, public officials, and private investors who lack urban cities’ reliable data on which to base crucial decisions. This also hinders future research and advocacy about the vital role of cities in local economic development and sustainable livelihoods. As a result, policymakers could not be held to account sufficiently for the lack of progress in strengthening local municipal economies and narrowing urban cities and regional information gaps for sustainable development (SEAD-SA 2023).

Poorly managed urbanisation and a lack of planning threatened people’s livelihoods, local economic development plans, environmental sustainability, and social equity (IFRC 2017). Most disaster management plans remain disconnected from the sectoral development plans. The plans are ignored because they are either not supported by sufficient resources or a lack the backing of an accountability framework, which can also serve as a DRRM framework for building urban resilience (UN (c) 2017). The response to climate-induced risks is predominantly at a local level. This renders local government relevant to implementing policies and strategies that lessen the impacts of climate-induced risks on individuals and communities (Williams et al. 2018). Although urban resilience is a preamble to be considered as a critical ingredient to achieving the vision set up for most global frameworks and global targets, such as the SFDRR, on issues such as local government roles, the SFDRR still lacks coherent guidelines on building urban resilience into the urbanisation system processes. The SFDRR also lacks coherent guidelines for specifying the roles and duties of multi-stakeholders in implementing DRR within developmental sectors. The SFDRR provides little information on how cities and urban governance of metropolitan shape their resource plans and needs, and what is possible and realistic to implement while building urban resilience.

Stuart et al. (2015) expressed the view that many of the required legislative are in place for local municipalities striving to end poverty, champion a sustainable human settlement environment, and foster healthcare and job opportunities with decent living wages for all people. However, developmental progress is impeded by a lack of community lower-level data because local governments do not adequately understand the living arrangements of their poor people. The international communities wouldn’t be able to support intervention programmes aimed at the marginalised and poor vulnerable communities without overhauling the current ways of gathering statistics (Stuart et al. 2015).

Faiella (2020) believes that having accurate data on sector-specific disaster losses and damage is a crucial indication for policymaking and DRR progress evaluation because of evidence-based provision of knowledge. Furthermore, records of losses and damage because of natural and man-made disasters within urban cities’ human settlements are not always available.

Gomez-Alvarez et al. (2021) supported the view that there is a global shift in conceptualising and understanding the cities’ resilience progress beyond economic metrics and towards an inclusive, comprehensive perspective that prioritises human and environmental well-being as the core point for developmental interventions. The shortcomings and inadequacies demonstrated by the conventional economic indicators as standards for development reveal that urban well-being can no longer be measured in terms of economic progress.

The urban DRR studies advocate for a multi-disciplinary stakeholder approach but tend to ignore the communities in high disaster-risk areas. Public–private partnership (PPP) seems to be non-existent. For instance, a study was conducted by Tun (2020) to develop an urban DRR framework. The objective of a study conducted by Sandoval, Sarmiento and Meenakshi (2020), was to understand the relationship between land security of tenure for dwelling ownership and DRR. The findings indicated that there is no significant causal relationship that by achieving security of land tenure, dwelling owners will start improving their properties for DRR mitigation. As a result, this study fills the gaps with the aim to use the concept of social resilience to envisage an outcome that intends to go beyond the local municipal urban cities’ communication and public participation advocacy strategies and extends the DRR information dissemination for in situ informal settlements.

## Research methods and design

### Study areas

**FIGURE 1:**
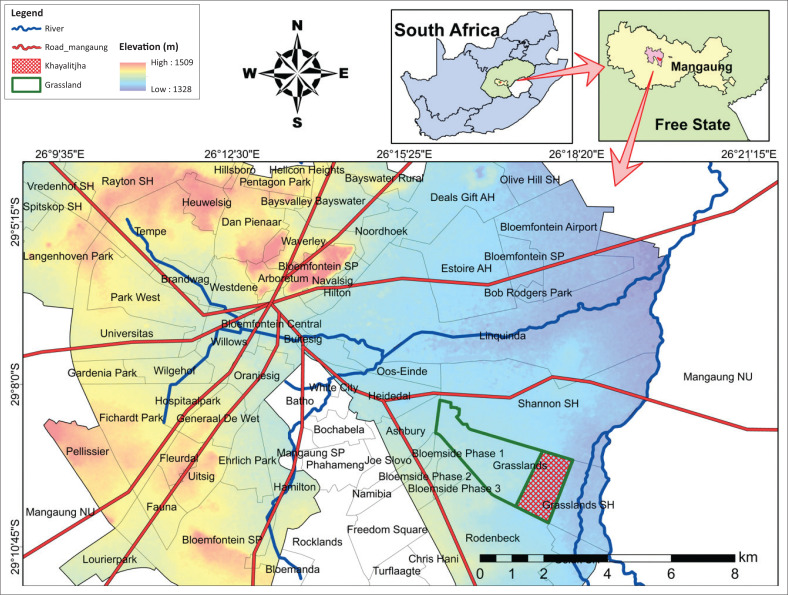
Khayalitjha in-situ informal settlement study area within Bloemfontein.

The location area for this study is commonly known as ‘Grassland Phase 3’ or Khayalitjha informal settlement in Bloemfontein, South Africa. Bloemfontein is also the sixth-largest city and judicial capital of South Africa and the capital city of the Free State Province (RSA MMM (h) 2021). The sole purpose for selecting Khayalitjha in situ informal settlement is because of the fact it is in an urban metropolitan city. Figure 1 depicts the geographical map of South Africa, the Free State province, and the location of the city of Bloemfontein within the Mangaung Metropolitan Municipality (MMM).

Khayalitjha used to be a privately owned agricultural small-holding land. It was developed into a residential area because of a land invasion in the late 1990s. Mangaung Metropolitan Municipality obtained the Grassland Eviction Order for the Housing Development Project for low-cost housing development. The land was invaded and occupied since June 2004, and eventually, the development of Grassland Phase 3 came to a dead end when homeless people invaded the agricultural smallholding land (Mphambukeli 2015).

Khayalitjha Census Geographical Area is based on 12 Enumeration Areas (EAs). These EAs are the lowest census geographical levels that assist the enumeration fieldworker in identifying both the location and the dwelling units for data collection. These EAs are made out of different settlement types. According to Statistics South Africa (Stats SA), the demographic profile (Stats SA (e) 2018) of Khayalitjha is informal settlement in Ward 17 with the current new municipal demarcation of MMM (RSA MMM (h) 2021). The community profile of Khayalitjha is as follows:

- Total population = 8319, of which 7973 are black African, 323 are mixed raced; 20 are Indian, and 4 are white.
- Total Households = 8305, of which 7923 reside in informal dwellings, 321 in formal dwellings, 60 in other types of dwellings, and 2 in traditional dwellings.
- It is composed mainly of ‘One-Person-Headed Households’, just like any other informal settlement in South Africa (Stats SA (e) 2018).

### Sampling procedure and method

The study is a cross-sectional household survey that used mixed modes of data collection. The Computer Telephone Interviews (CATIs) and the Paper Assisted Personal Interview (PAPI) are the two modes of data collection used for the household-based survey at Khayalitjha in situ informal settlement. The overall sample size was fixed at 5% of the total sample frame (i.e. 8305 household estimates), with a total sample size of about 415 household heads drawn to voluntarily participate in the study, of which 295 households responded in 2021. A quantitative method was employed in this study. Community Preferred Process Facilitator and Community Preferred Mode of Communication respond to social resilience, measured by three indicators:

1. Secure security of dwelling unit tenure: The household head is assigned the plot stand and plot number by the local municipality and occupies the dwelling unit free from paying rent.
2. Insecure security of dwelling unit tenure: The household head is renting the dwelling unit from the landlord within an assigned plot by the local municipality, and
3. No secure security of dwelling unit tenure: Household head occupied dwelling unit not located within the demarcated local municipal assigned plot or stand irrespective of paying or not paying rent from the landlord.

### Ethical considerations

Ethical clearance to conduct this study was obtained from the University of the Free State General/Human Research Ethics Committee (GHREC). (No. UFS-HSD2021/0660/21).

## Results and discussion

The main objective was to determine the participation and communications of Khayalitjha in situ informal settlement residence regarding community participation in DRR and in situ informal settlement upgrading. As a result, the household heads of the Khayalitjha informal settlement were asked to identify within their community who participated in developing the disaster plan. Table 1 indicates the household heads’ response to the community participation question regarding disaster preparedness.

**TABLE 1:** Community participation for disaster preparedness through social resilience indicators (secure tenure, in-secure tenure, and no-tenure).

Community	Social resilience						Total	
	Secure tenure		In-secure tenure		No-tenure			
	*n*	%	*n*	%	*N*	%	*n*	%
Ward Councillor	33	11	6	2	17	6	56	19
Ward Committee Member	15	5	4	1.4	68	23	87	29
Community Police Forum Member	8	2.7	47	16	2	0.7	55	19
Community Volunteers	4	1.4	54	18.3	37	12.5	97	32

**Total**	**60**	**20.1**	**111**	**37.7**	**124**	**42.2**	**295**	**-**

Almost one-third of the household heads identified Community Volunteers (32%) and Ward Committee members (29%) as active participants in developing disaster preparedness plan through social resilience indicators. This indicates that the legislative structures, such as the Ward Councillors and the Community Police forums, need to be strengthened to gain the trust of informal settlement communities.

This finding aligned with *The South African Promotion of Access to Information Act 2 of 2000*, intended to give effect to the people’s constitutional right of access to any information held by the government and information held by any other person and that is required for the exercise or protection of any rights, and also to provide for matters connected after that. Community participation of informal settlement residents should thus be strengthened and supported by the local municipality by conducting regular household-based surveys as an evidence-based public consultation for community needs identification regarding DRRM.

The household heads were further asked to name who should be responsible for starting the process to reduce disaster risks and improve disaster preparedness within the community. Their responses are quantified in Table 2.

**TABLE 2:** Community preferred process facilitator for disaster preparedness by social resilience.

Community	Social resilience						Total	
	Secure tenure		In-secure tenure		No-tenure			
	*n*	%	*n*	%	*n*	%	*n*	%
Ward Councillor	29	10	46	16	15	2.1	90	31
Ward Committee Member	11	4	41	14	59	20	111	38
Community Development Worker	12	4	5	1.7	14	4.7	31	10.4
NGOs, NPOs, CBOs	4	1.4	0	0	12	4.1	39	5.5
Provincial Disaster Management Centre Representative	1	0.3	12	4	11	3.7	24	8
District Disaster Management Centre Representative	3	1	7	2	13	4	23	7

**Total**	**60**	**20.7**	**111**	**37.7**	**124**	**41.6**	**295**	**-**

NGOs, non-governmental organisations; NPOs, non-profit organisations; CBOs, community-based organisations.

The Ward Committee members and the Ward Councillors were preferred by one-third (i.e. 38% and 31%, respectively) of household heads to be responsible for initiating the DRR and disaster preparedness process within the informal community. This is a positive move because both Ward Councillors and Ward Committees are legislated and will easily receive support from the local municipality. The policy implications informed by the research findings of Ward Committee members and Ward Councillors, both strongly preferred by one-third of informal settlement households of Khayalitjha, point the local government towards the need for human resource capacity building for DRRM information dissemination at a local level. A similar study was conducted by Williams (2015) by reviewing and assessing community participation in practice, drawing on the findings of a range of research projects conducted in Cape Town. According to this study, the presence of ordinary people in local government structures presupposes the requisite political space to challenge the uneven power relations at the local community level and elsewhere. Individualistic participation ambitions can override and undermine the common community objective.

Table 3 explored the preferred mode of communication for disaster information dissemination. It is of interest to observe that household heads identified the top five preferred ways of communication for early warnings and disaster information dissemination to be as stated: Church/Schools (21%), WhatsApp (i.e. 16%), Facebook/Twitter/Instagram (i.e. 11%), Community radio (i.e. 11%), and the distributions of pamphlets and mounting of posters (i.e. 9%). It is evitable from the research findings that the South African economy is digitalising rapidly, and communities at urban informal settlements are also ready to embrace this new paradigm shift. This simply means that urban city citizens and consumers, from either private or public sectors, will access most of the services on digital platforms through smartphones, tablets, or any accessible electronic devices. Most South African government departments and private sector organisations are transforming their service delivery model by shifting them to a digital domain (RSA MMM (h) 2021), through the development of the urban resilient DRRM policy framework that harnesses the economic and social potential of data and cloud computing because of digitalisation.

**TABLE 3:** Community-preferred mode of communication for disaster information dissemination.

Community	Social resilience						Total	
	Secure tenure		In-secure tenure		No-tenure			
	*n*	%	*n*	%	*n*	%	*n*	%
WhatsApp	16	5	14	5	17	6	47	16
Facebook/Twitter/Instagram	3	1	20	7	10	3	33	11
Community Radio	9	3	17	6	5	2	31	11
Mainstream Radio	1	0.3	15	5	8	3	24	8.3
Pamphlets and Posters	3	1	6	2	19	6	28	9
Loudspeaker	9	3	0	0	6	2	15	5
Community meetings	6	2	11	4	0	0	17	6
Church/Schools	1	0.3	10	3	52	18	63	21
Public Service Outlet	8	3	8	3	4	1	20	7
Television	4	1.4	10	3	3	1	17	5.4

**Total**	**60**	**20**	**111**	**38**	**124**	**42**	**295**	**-**

## Conclusion

Community volunteers and ward committee members have the lion’s share as active participants and are preferred for responsibly initiating the process of DRR and disaster preparedness plans. Church and school were the first preferred mode of communication for early warnings and disaster information dissemination. It is concluded that the legislative structures, such as the Ward Councillors and the Community Police forums, must be empowered to gain trust from informal settlement communities.

The key findings complement the National Upgrading Support Programme (NUSP) to fill a critical gap in technical support, capacity building, and sharing lessons learned. The key findings also advocate for a strong emphasis on DRR planning with the intention of what developers and planners aim to change together with the societal impact of both metropolitan and non-metropolitan municipalities’ interventions.

The study further recommends that local government needs to enhance human resource capacity building for DRRM information dissemination at a local level. The legislative structures need to be strengthened to gain the trust of informal settlement communities. Last but not least, the community needs to embrace the new paradigm shift of rapid digitalisation.
